# Unusual late-fall wildfire in a pre-Alpine *Fagus sylvatica* forest reduced fine roots in the shallower soil layer and shifted very fine-root growth to deeper soil depth

**DOI:** 10.1038/s41598-023-33580-7

**Published:** 2023-04-19

**Authors:** Antonio Montagnoli, Mattia Terzaghi, Alessio Miali, Donato Chiatante, R. Kasten Dumroese

**Affiliations:** 1 Department of Biotechnology and Life Science, University of Insubria, Via Dunant, 3, 21100 Varese, Italy; 2 Department of Biosciences, Biotechnologies and Environment, University of Bari Aldo Moro, Piazza Umberto I, 70121 Bari, Italy; 3 Rocky Mountain Research Station, U.S. Department of Agriculture Forest Service, Moscow, ID USA

**Keywords:** Ecology, Climate-change ecology, Fire ecology, Forest ecology, Plant ecology, Plant stress responses, Abiotic

## Abstract

After an unusual, late-fall wildfire in a European beech (*Fagus sylvatica* L.) forest in the pre-Alps of northern Italy, the finest roots (0‒0.3 mm diameter) were generally the most responsive to fire, with the effect more pronounced at the shallowest soil depth. While roots 0.3‒1 mm in diameter had their length and biomass at the shallowest soil depth reduced by fire, fire stimulated more length and biomass at the deepest soil depth compared to the control. Fire elevated the total length of dead roots and their biomass immediately and this result persisted through the first spring, after which control and fire-impacted trees had similar fine root turnover. Our results unveiled the fine-root response to fire when subdivided by diameter size and soil depth, adding to the paucity of data concerning fire impacts on beech roots in a natural condition and providing the basis for understanding unusual fire occurrence on root traits. This study suggests that *F. sylvatica* trees can adapt to wildfire by plastically changing the distribution of fine-root growth, indicating a resilience mechanism to disturbance.

## Introduction

Fine roots of woody plants are commonly defined as a single pool of short-lived, non-woody, mycorrhizae-associated roots less than 2 mm in diameter^1–4^. Their function of absorbing water and nutrients is crucial to plant survival potential^1^. While fine roots rarely represent more than 5% of total biomass, their annual production amounts to 33–67% of the total annual net primary production in most terrestrial ecosystems^5^. Thus, fine roots significantly influence biogeochemical processes, and their dynamics have a central role in the global carbon budget^6,7^. Moreover, the total fine root biomass (living fine roots) and necromass (dead fine roots) reflect overall fine root production, death, and decomposition and can inform how trees interact with their environments and provide ecological functions^5,8^.

Within the total root system, fine roots are the most sensitive and dynamic component, rapidly responding to rooting environment perturbations^9,10^. Moreover, fine root biomass and length seasonally fluctuate due to both endogenous (e.g., genotype of plant species) and exogenous (e.g., temperature, precipitation, soil properties, nutrient availability, and competition among plants) factors^2^. Fine root dynamics are strongly affected by disturbances^11^ including fire^12,13^.

Fine root response to fire varies. On one hand, annual production of fine roots is expected to increase because of the need to recuperate lost root function^14^. On the other hand, carbon supply for root growth could be limiting, particularly if a high degree of canopy loss occurred^15^. Also, surface fire influences fine root dynamics directly because fire-derived heat substantially reduces root biomass and specific root length^13,14^. Fine root mortality relates to the amount of soil heating, a function of forest floor moisture content and fire intensity^16,17^, and root depth distribution^13^.

Although wildfire in Europe is usually associated with the fire-prone countries surrounding the Mediterranean Sea, the alpine region of Central Europe experiences wildfires of relatively low frequency, intensity, and size that mostly (~ 90%) occur on southern slopes of the Alps^18–20^. Historically, these fires occurred either in early spring (March and April) or summer (July and August)^21^. A long-term analysis (1951–2010) of indices of forest fire danger and ten years of observed forest fires (2001–2010) revealed a significant increase in the western Alps and an even stronger increase in the southern Alps^22^, and fire regimes are expected to react dynamically to alterations in the climate–weather–fuel system with changes in fire intensity, seasonality, frequency, and scale^23,24^. Indeed, extraordinary changes in alpine fire regimes have been observed^25^, often associated with temporal climate variability^26–28^, and especially with heat waves and dry foehn winds, which are generally regarded as an indication of a changing climate that will lead to new fire regimes in the Alps^21,29,30^.

Each ecosystem is adapted to a specific fire regime^31^; thus, the rise of new wildfire regimes, which implicate an increase in occurrence and severity, may cause abrupt changes in the functioning of forest ecosystems^32–34^. This may be especially true for European beech (*Fagus sylvatica* L.; hereafter beech) forests in the southern Alps that experienced exceptionally numerous and large fires during the hot and dry summer of 2003^29^. Beech, like most tree species growing in the Alps and Central Europe, lacks obvious fire resistance or adaptation traits such as thick bark, strong resprouting ability, serotiny, or a smoke germination cue. Mature beeches are considered highly susceptible to fire^35^. Notwithstanding, recent studies indicate that low to moderate burn severity increases the survivability of seed-providing beech trees; the result is favorable short-term germination conditions that initiate rapid beech regeneration processes^36^. On the contrary, severe fires cause early deaths in beech trees, which may inhibit beech regeneration^29^. In sum, when a fire regime deviates from expected patterns, the resilience of the ecosystem to fire may be exceeded, putting in danger the ability of species within that ecosystem to survive and regenerate.

In 2017, in the western part of the Italian Pre-Alpine belt, wildfire deviated from the expected timing patterns (i.e., summer season) and occurred in late October. The fire was atypical because, historically, August–September precipitation diminishes fire activity, but, a long-term spring–summer drought persisted, allowing a fire to ignite in late October and burn until early November in pure beech forests. Because of the paucity of information about fire effects on beech fine roots (i.e., spatial and temporal), the novel timing of this fire, the need to better understand the repercussions of novel fire regimes, and because fine roots are a good indicator of forest adaptation to climate change^37,38^, we initiated a study to examine fire effects on beech fine roots. Our objective was to understand how beech trees modify the growth of their fine roots to adjust to changes in the rooting environment caused by an unusual late fall wildfire. We hypothesized that late fall unusual wildfire would lead to functional differentiation of seasonal and spatial modulations of fine roots in beech trees.

To test our hypothesis, we selected burned (moderate severity) and unburned beech trees after the 2017 autumnal wildfire and used standard soil-coring techniques to obtain and measure fine root dynamics immediately (one month after the fire) and during the next two growing seasons. Unveiling these fine-root characteristics may enhance the understanding of the resilience of beech forests to fire and provide insights on functional responses to stress determining plant survival and growth.

## Material and methods

With permission of local authorities of the Regional Natural Park ‘Campo dei Fiori’ in the Lombardy Prealps region of northern Italy, we non-lethally collected roots of beech, a common, widespread species across Europe that is not listed as threatened or endangered in the IUCN list (indicated as having the ‘least concern’ status in the Red List Assessment; https://www.iucnredlist.org/species/62004722/80570512).

Roots were collected carefully using soil cores to minimize forest floor disturbance. Mount Campo dei Fiori (1227 m a.s.l.) lies within a regional nature park in the Lombardy Prealps region of northern Italy. On this mountain, mixed forests (*Castanea sativa* Mill., *Fraxinus excelsior* L., *Tilia cordata* L., *Acer pseudoplatanus* L., and *Corylus avellana* L.) dominate below 600 m a.s.l. with pure beech forests above 600 m. The area experiences a sub-continental temperate climate having mean annual precipitation of 1500–2000 mm concentrated in April–May and August–September and mean annual temperature of 10–14 °C^39,40^. A 243-ha wildfire burned on southern aspects in late October—early November 2017 after leaves had senesced. In-situ post-fire surveys revealed a range of fire intensities and subsequent effects on soil and understory and overstory vegetation^41^.

**Figure 1:**
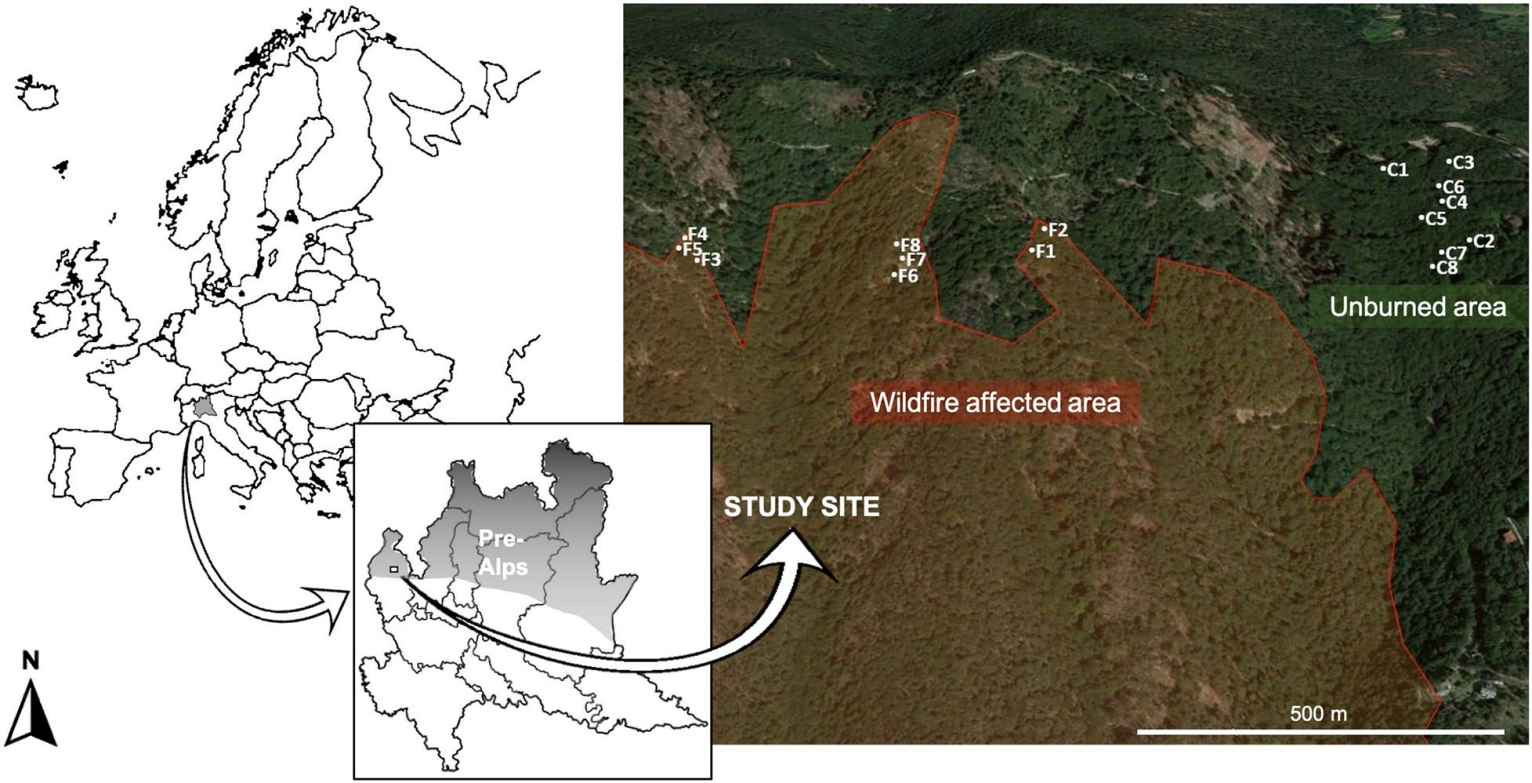
Map of the study site (Pre-Alps of the Lombardy region Northern Italy) reporting the wildfire-affected area and the unburned area with, respectively, the selected burned (F) and control (C) trees. The maps included in the figure were captured from TENtec Interactive Map Viewer—European Commission and from Geoportale della Lombardia, modified and assembled with ImageJ 1.53a (Wayne Rasbanb, National Institute of Health, USA; https://imagej.nih.gov/ij/download.html) and PowerPoint for Mac 16.60 (Microsoft® corporation; www.microsoft.com) software, respectively. Authors A.Mo, A.Mi, and M.T. created the figure.

We selected eight fire-affected and eight control (unburned) beech trees from, respectively, a moderate burn severity area^41^ and an unburned area about 500–1000 m apart at comparable elevations (Fig. 1; ~ 1100 m a.s.l.; center point latitude 45.86 N, longitude 8.77 E), and having similar ranges and mean values of stem diameter at breast height (Table 1).

**Table 1:** Height^a^ and diameter breast height (DBH)^b^ of fire-affected and control beech trees.

Control area (unburned)			Fire-affected area		
Tree ID	DBH (cm)	Height (m)	Tree ID	DBH (cm)	Height (m)
C1	29.0	15.0	F1	46.3	16.9
C2	21.1	12.6	F2	58.4	20.4
C3	51.3	20.3	F3	35.3	14.3
C4	71.0	21.8	F4	45.4	18.5
C5	24.8	14.8	F5	61.0	18.8
C6	67.0	23.2	F6	98.9	24.4
C7	45.3	15.9	F7	30.0	16.0
C8	57.4	19.4	F8	21.9	14.6
Mean	45.9 ± 6.4	17.9 ± 1.3	Mean	49.7 ± 7.9	18.0 ± 1.1

^a^Laser measured (TruPulse® 200; Laser Technology, Inc.; Centennial, CO, USA.

^b^Measured approximately 130 cm above groundline.

Individual trees were within 30–50 m of their nearest neighbor, all trees (burned and control) grew within a 1000-m-long transect and were considered independent replicates. Average slope is ~ 46%. The thin, clay-loamy soil^41^ is a leptic and eutric Cambisol^42,43^. We sampled trees one month after the wildfire (December 2017), in May, July, and October of 2018, and in July 2019. Of the eight burned trees, only six were subsequently resampled because one fell down and another had poor (< 20%) canopy recovery. For each tree and sampling time, we randomly collected two soil cores (4 cm diameter × 30 cm deep) 70–100 cm from the stem using a motor-driven, portable soil core sampler. Cores were stored in plastic bags at 4 °C until processed (within 20 days of collection).

Each 30-cm soil core was divided into three 10-cm segments for processing. Briefly, each segment was enclosed in a nylon bag (300 μm mesh) and washed with cold water in a laundry washing machine until the soil was sieved (only roots and small rocks remained). Using tweezers, we removed all fine roots (diameter ≤ 2 mm) and with the aid of a stereomicroscope (Nikon SMZ 800) divided them into two main groups: *Fagus sylvatica* and other species. The area is characterized by an occasional presence of the shrub *Corylus avellana* and the tree *Fraxinus excelsior*, which were, when present, growing at minimum distance of 6‒8 m from the selected beech trees. This reduced the presence of fine roots other than beech in our soil core samples. To ensure accurate distinguishment of beech fine roots from those of other species, we collected fine root samples for all fine root categories of *F. sylvatica, C. avellana, and F. excelsior* by carefully digging and tracking roots to each species. These roots were analyzed in their appearance, morphology, and anatomy to provide a precise distinction of the fine roots. Beech fine roots were reddish colored both in the rhizodermis characterizing the primary body of the smaller diameter fraction (0‒0.3 mm) and in the periderm characterizing the secondary growth of larger diameter fine roots. We also used, when root origin was in doubt during the process, a transversal cut on fresh root material was carried out to exactly establishing the plant species’ fine root. The fine roots of herbaceous species were easy to distinguish due to their clearly different structure and texture compared to the woody plants.

Beech fine roots were segregated into live (biomass) and dead (necromass) by color, texture, turgor, and shape. We scanned roots at 800 dpi using a calibrated flatbed scanner coupled to a lighting system for image acquisition (Epson Expression 10,000 XL). We analyzed subsequent images using software (WinRhizo Pro V. 2007d, Regent Instruments Inc. Quebec, Canada) to separate roots into fine (< 2 mm) and coarse (> 2 mm) diameter (d) categories. We separately oven-dried live and dead roots (60 °C to constant mass) independently to obtain their biomass and necromass.

Fine root morphological traits such as length (mm) and biomass (g) were measured within three diameter size sub-classes (d < 0.3 mm, Class 1 (hereafter C1); 0.3 < d < 1.0 mm, C2; 1–2 mm, C3) and are expressed per m^2^ and a defined soil depth. Fine root biomass for the entire 0–2 mm diameter was considered total dry mass. To calculate biomass values for each diameter sub-class, root volume (cm^3^) for C1, C2, and C3 roots was multiplied by 0.35, 0.49, and 0.78 g·cm^−3^ tissue density, respectively. The C1 value was the mean of the range (0.25–0.45) reported by Beyer et al.^44^ and C2 and C3 are the lowest and highest values, respectively, of the range (0.49–0.78) published by Hertel et al.^45^.

We estimated annual fine root production for each beech using live and dead fine root patterns. The delta of live and dead root mass was evaluated between each sampling time from December 2017 to October 2018 using the decision matrix method^46,47^. Mean standing biomass and necromass were calculated as the average of annual live and dead fine root standing crop, respectively. We calculated fine root turnover rates of live biomass as annual root production divided by the maximum standing biomass^48^. Live to dead mass ratio was calculated as the live mean standing biomass to the dead mean standing necromass ratio. We defined specific root length as the ratio of root length to root mass^49^.

### Statistical analysis

Data from the two soil cores obtained from each tree were averaged before proceeding with statistical analysis. For each fine root diameter class (C1, 0–0.3; C2, 0.3–1.0; C3, 1–2 mm), hierarchical linear regression (HLR) analysis was applied to live and dead root mass, length, and specific root length to investigate whether predictor variables (sampling time, fire, soil depth) were dependent on the soil core from which root traits data were obtained (grouping variable). The results of HLR showed that the exclusion of the grouping variable reduced the models predictiveness by 2.4 ± 1.6% (means ± SD, % R squared variation in Supplementary Table S1). Therefore, we proceeded in our analysis treating fire, sampling time, and soil depth as independent factors. We employed a three-way ANOVA for each fine root diameter class (C1, C2, and C3) to examine the independent variables of fire, sampling time, and soil depth factors on the dependent variables of live and dead root mass, length, and specific root length. Additionally, for production and turnover rate, we employed a two-way ANOVA to examine the independent variables of fire and soil depth. As needed, we used square root or log to transform dependent variables to meet normal distributions and equal variances. To compare differences between burned and non-burned trees paired t-tests were performed. We analyzed data using statistical software (SPSS 25.0, SPSS Inc., Chicago, IL, USA) and considered differences significant at *p* < 0.05.

### Ethical policies

The authors declare that the collection and use of plant materials in this study are carried out in compliance with the IUCN Policy Statement on Research Involving Species at Risk of Extinction and the Convention on the Trade in Endangered Species of Wild Fauna and Flora. In particular, a non-lethal collection of plant root material was performed in a European beech (*Fagus sylvatica* L.) wild forest; this species, along with *Corylus avellana* and *Fraxinus excelsior* are listed with ‘least concern’ status in the Red List Assessment of the IUCN. The material authors required was unavailable in a museum or other institutional collections. Finally, the authors collected the minimum number of specimens necessary to accomplishment of their research.

### Voucher specimen

Voucher root specimens for all wild sampled plants of the present manuscript are deposited in a collection owned by the public University of Insubria at the Laboratory of Environmental and Applied Botany (via Monte Generoso, 71–21100 Varese, IT), which provides public-free access to the material. The voucher specimens analyzed in the present work were identified by Antonio Montagnoli and consist of European beech dry fine-root fragments of different lengths, diameter classes, soil depths, and individuals.

## Results

In all analyses, the three-way interaction (fire × sample time × soil depth) was not significant (*p* ≥ 0.8); specific *p* values are omitted from the results.

### Live root length, biomass, and specific root length

**Figure 2:**
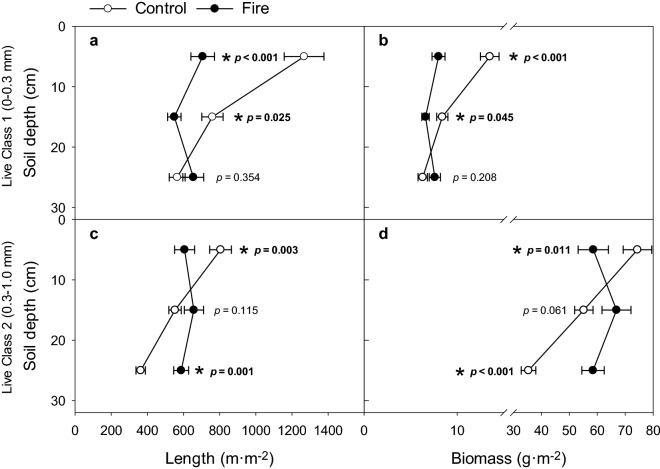
The significant interactions of fire × soil depth on live fine root length (m·m^−2^) and standing biomass (g·m^−2^) for Root Class 1 (0–0.3 mm) (**a)** and (**b)** respectively) and Root Class 2 (0.3–1.0 mm) (**c**) and (**d**) respectively). For control (○; n = 40) and fire-affected (●; n = 30) trees, significant differences (*p* < 0.05) determined by paired t-test and shown in bold with an asterisk.

The interaction of fire and soil depth was significant for live root length and biomass for C1 (0–0.3 mm) and C2 (0.3–1.0 mm) roots (Table 2; Fig. 2).

**Table 2:** Statistics (F and *p* values) from three-way ANOVAs for each fine root diameter class for the independent variables fire, sampling time (time), and soil depth, and the fire × time and fire × soil depth interactions on the dependent variables live root length, live root biomass, dead root length, necromass, and live specific root length; and statistics (F and *p* values) from two-way ANOVAs for each fine root diameter class for the independent variables fire and soil depth and the fire × soil depth interaction for the dependent variables annual production and turnover.

Variables	Root diameter (mm)					
	Class 1		Class 2		Class 3	
	0–0.3		0.3–1.0		1.0–2.0	
	F	*p* value	F	*p* value	F	*P* value
Live length						
Fire (F)	33.01	** < 0.001**	2.17	0.141	0.03	0.854
Time (T)	7.56	** < 0.001**	4.84	**0.001**	14.98	** < 0.001**
Soil depth (D)	145.89	** < 0.001**	166.97	** < 0.001**	120.66	** < 0.001**
F × T	0.04	0.996	3.73	**0.005**	1.71	0.147
F × D	7.84	** < 0.001**	5.30	**0.001**	0.18	0.908
Live biomass						
Fire (F)	14.07	** < 0.001**	2.77	0.097	0.01	0.944
Time (T)	5.33	** < 0.001**	2.85	**0.024**	10.00	** < 0.001**
Soil depth (D)	13.70	** < 0.001**	10.17	** < 0.001**	4.46	**0.012**
F × T	0.25	0.911	2.40	**0.049**	0.84	0.500
F × D	11.64	** < 0.001**	13.17	** < 0.001**	0.48	0.617
Dead length						
Fire (F)	90.91	** < 0.001**	52.78	** < 0.001**	11.59	** < 0.001**
Time (T)	52.18	** < 0.001**	78.15	** < 0.001**	10.02	**0.002**
Soil depth (D)	27.75	** < 0.001**	37.20	** < 0.001**	35.85	** < 0.001**
F × T	17.36	** < 0.001**	15.15	** < 0.001**	4.85	**0.001**
F × D	3.49	**0.016**	5.53	**0.001**	1.09	0.353
Necromass						
Fire (F)	45.78	** < 0.001**	54.30	** < 0.001**	7.53	**0.006**
Time (T)	113.19	** < 0.001**	41.35	** < 0.001**	6.83	** < 0.001**
Soil depth (D)	16.33	** < 0.001**	10.88	** < 0.001**	4.49	**0.012**
F × T	8.82	** < 0.001**	5.06	**0.001**	3.40	**0.010**
F × D	1.00	0.368	0.17	0.842	0.96	0.383
Specific root length						
Fire (F)	27.201	** < 0.001**	4.725	**0.030**	0.325	0.569
Time (T)	5.018	**0.001**	3.845	**0.005**	3.378	**0.010**
Soil depth (D)	3.789	**0.024**	6.481	**0.002**	0.732	0.482
F × T	0.814	0.517	2.146	0.075	1.275	0.280
F × D	0.779	0.460	0.028	0.973	3.027	**0.049**
Production						
Fire (F)	0.258	0.615	0.934	0.342	2.025	0.165
Soil depth (D)	1.967	0.157	1.196	0.316	0.051	0.950
F × D	0.386	0.683	1.232	0.306	0.717	0.497
Turnover						
Fire (F)	2.379	0.133	0.263	0.612	2.146	0.153
Soil depth (D)	3.747	**0.035**	0.745	0.483	0.869	0.430
F × D	0.606	0.552	1.063	0.358	1.471	0.246

Significant values are in bold.

**Figure 3:**
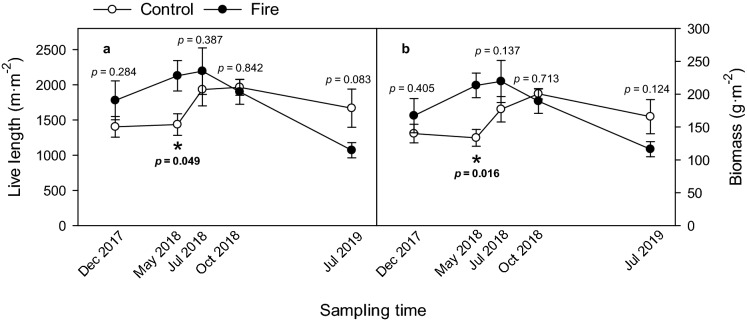
The significant interactions of fire × sampling time on live fine root length (m·m^−2^) (**a**) and standing biomass (g·m^−2^) (**b**) for Root Class 2 (0.3–1.0 mm). For control (○; n = 8) and fire-affected (●; n = 6) trees, significant differences (*p* < 0.05) determined by paired t-test and shown in bold with an asterisk.

No interactions were significant for C3 (1.0–2.0 mm) roots. For C1 roots, fire was significant for length and biomass in the two shallowest soil profiles (0–10 and 10–20 cm) but not at the deepest profile (20–30 cm) (Fig. 2a,b); control trees had longer live root length and more biomass. For C2 roots, fire was significant for length and biomass in the shallowest (0–10) and deepest (20–30) soil profiles but not in the mid-profile (Fig. 2c,d). For C2 roots, burned trees had less live root length and biomass than controls at the shallowest depth, but at the deepest soil depth, the results were opposite (Fig. 2c,d). For C2 roots, fire interacted with sampling time to affect live root length and biomass (Table 2). In the spring following fire (May 2018), fire-affected trees had longer live root length and more biomass than the control (Fig. 3a,b).

Fire was significant only for C1 root length and biomass (Table 2); summed across depths and averaged through the sampling period, fire-affected trees had less live root length (1909 vs. 2592 m·m^−2^) and less biomass (22.2 vs. 28.2 g·m^−2^) compared to the control (Supplementary Table S2). Soil depth, for control and fire-affected trees combined, was significant for live root length and biomass and all root diameter classes (Table 2); the sums of lengths significantly decreased as depth increased from 0–10 to 10–20 to 20–30 cm (Supplementary Table S2). For C1 roots, fire, time, and soil depth variables were significant for specific root length (Table 2); the values were always larger for control vs. fire-affected trees, and varied by depth and sampling time (Supplementary Table S2).

### Dead root length and necromass

**Figure 4:**
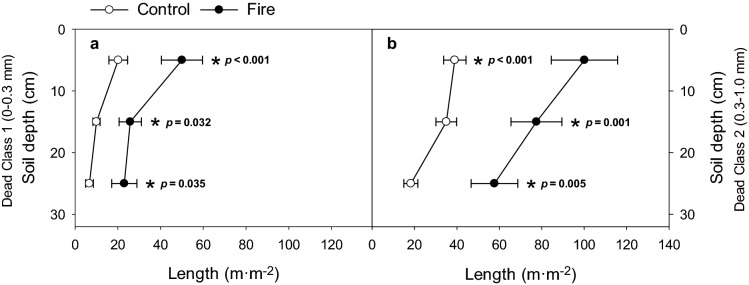
The significant interactions of fire × soil depth on dead fine root length (m·m^−2^) for Root Class 1 (0–0.3 mm) (**a**) and Root Class 2 (0.3–1.0 mm) (**b**). For control (○; n = 40) and fire-affected (●; n = 30) trees, significant differences (*p* < 0.05) determined by paired t-test and shown in bold with an asterisk.

The interaction of fire and soil depth was significant for dead root length for C1 (0–0.3 mm) and C2 (0.3–1.0 mm) roots (Table 2) but not for necromass. The fire × soil depth interaction was not significant for either dependent variable in C3 (1.0–2.0 mm) roots. For C1 and C2, dead root length was significantly higher in burned trees for the three soil depths analyzed (Fig. 4), and the difference in length between burned and unburned trees was greatest at the shallowest soil depth.

**Figure 5:**
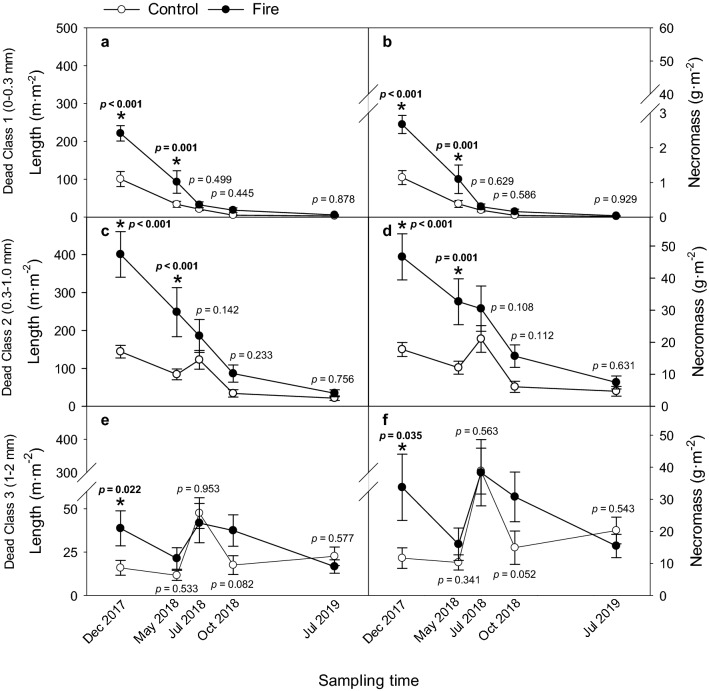
The significant interactions of fire × sampling time on dead fine root length (m·m^−2^) (**a**, **c**, and **e**) and standing necromass (g·m^−2^) (**b**, **d**, and **f**) for Root Class 1 (0–0.3 mm; a and b), Root Class 2 (0.3–1.0 mm; c and d), and Root Class 3 (1.0–2.0 mm; e and f). For control (○; n = 8) and fire-affected (●; n = 6) trees, significant differences (*p* < 0.05) determined by paired t-test and shown in bold with an asterisk.

For all three root classes, the fire × sampling time interaction was significant for dead root length and necromass (Table 2). Compared to the control, we observed more necrotic roots immediately after fire on burned trees, and this effect persisted depending on root size and the depth where those roots were growing. For the first sampling time (Dec 2017) fire-affected trees had greater lengths of dead roots and more necromass than the control (Supplementary Table S2; Fig. 5), and for C1 and C2 roots, this pattern persisted through the second sampling time (May 2018). For all root classes, the length of dead roots and the amount of necromass decreased across the entire sampling period; the lowest values were observed in the C1 roots (Fig. 5). For C3 roots, values were more variable across time (Fig. 5). For all the root classes, and in particular for C2 and C3, dead root length and necromass in control trees showed a summer peak in July 2018 that was not detected during the next summer (July 2019) (Fig. 5).

Fire-affected trees had about 2.5 times more length and necromass at each soil depth for C1 and C2 roots compared to the control; length and necromass for these roots decreased with increasing soil depth (Supplementary Table S2). Fire-affected trees had more dead root length (15.0 vs. 8.8 m·m^−2^) and necromass (13.4 vs. 7.0 g·m^−2^) for C3 roots in the shallowest soil depth (0–10 cm) than the control; values of the control and fire-affected trees were similar at the other soil depths (Supplementary Table S2).

### Biomass ratio, fine root production, and turnover rate

The effect of fire on the ratio of live to dead biomass was significant at every soil depth for C1 roots (Supplementary Table S3); control trees had a higher ratio of live mass to necromass than fire-affected trees. The control ratio remained constant (81–102) at every soil depth, but the ratio for fire-affected trees increased (i.e., more live mass to necromass) with increasing depth (15.8–35.1–45.7; Supplementary Table S3). For C2 roots, the control ratio exceeded that of the fire ratio (16.0 vs. 5.2; Supplementary Table S3), but only at the shallowest depth was fire a significant effect. When considering all fine roots (0–2 mm in diameter) combined, fire was significant for standing necromass and dead length (Supplementary Table S2). For the entire 30 cm core, fire-affected trees compared with control trees had more necromass (55.9 vs 32.8 g m^−2^) and dead root length (288 vs. 138 m m^−2^).

## Discussion

The effects of global climate change on vegetation in general, and on root systems in particular, continue to challenge our comprehension^50^. In forest ecosystems, up to 60% of fine roots (< 2 mm diameter) occur in the upper 30 cm of soil^7^. The dynamic shifts in abundance of these roots can help inform how trees interact with their environment because fine roots constitute a substantial amount of annual plant biomass production, are responsible for water and nutrient uptake, and respond rapidly to fluxes in environmental conditions, including stochastic disturbances^34,11^. To better understand ecosystem resilience, we also need to better understand how various stressors, including for example, climate, drought, and fire, independently and in concert affect growth and development of single trees and their complex organization into forests^34^. Thus, in this study, we assessed fine root functional traits, such as length, biomass, specific root length, production, and turnover rate, in a pre-alpine beech forest recovering from autumnal wildfire at a novel time. Although we have no pre-disturbance measures, our first sample of live and dead roots by diameter and soil depth, taken in December one month after wildfire of moderate intensity and followed by sequential sampling during two consecutive growing seasons, provides a detailed insight into the effect of fire on fine root dynamics.

In our study, in support of the hypothesized seasonal differentiation, fire had temporal effects on fine roots; these affects varied with soil depth and whether or not the roots were alive or dead. Fire killed roots across all root classes with diminishing effects over time, suggesting an overall fine root lethal heating effect^13^. This effect may be observed at the lowest range of soil temperature from fires (50‒70 °C^51^), which in our case was differentiated based on the fine root diameter and soil depth distribution.

Indeed, while fire increased dead length and necromass across all root classes at the shallowest soil level (0–10 cm), only the smallest roots (C1 and C2) were affected in the deeper soil profiles (10–20 and 20–30 cm), with the effect diminishing with depth. Our observation is likely related to the depth of soil that reached lethal temperature, driven by fire intensity, heating duration, and fuel characteristics^52^. Furthermore, our root data allow us to infer that, although of lower magnitude, the soil temperature rise occurring closer to the soil surface affected thin roots mostly characterized by a primary structure (i.e., lacking bark) more than thicker roots (1–2 mm, C3), mainly devoted to water transport and possessing a periderm composed of cork tissue^53^. Periderm protects plant tissues from physical and biotic damage^54^. Finally, the lower magnitude of necromass detected in summer 2019 compared to summer 2018 could be due to the different fine root dynamics related to changes in environmental conditions^10^. Indeed, the high fine-root mortality measured in July 2018 might reflect the prolonged drought trees experienced during 2017 that rendered our study forest ecosystem more susceptible to wildfire.

This contrasts with live roots, where the temporal effect of fire, manifested as an increase in length and biomass, was only significant for C2 roots and only during the spring following fire. These findings might be related to a higher level of responsiveness to fire disturbance of these very fine roots (0.3‒1 mm), which include root types devoted to exploring and creating a framework (pioneer) and adsorbing nutrients (fibrous)^40^. These roots in burned trees, however, showed a descendent pattern after the initial spring increase, highlighting how fire may initially stimulate root production in response to heat-induced injuries (i.e., wounding) and/or flush of soil nutrient increase^53,55–57^, but subsequently have an opposite effect on root production. Moreover, the effect of fire on live root length and biomass averaged on the entire sampling period was inconsistent by depth but showed a clear reduction of fine roots, especially in the shallower soil layers. Regardless of root class, control fine roots decreased with increasing soil depth (the expected forest ecosystem response) while fire inverted this pattern, stimulating a higher production of fine roots deeper in the soil profile. For C1 roots, fire reduced length and biomass the most at the two shallowest levels, with more reduction closer to the soil surface. For C2 roots, however, increasing depth revealed a transition where fire reduced the length and biomass near the soil surface but increased root biomass and length at the deepest level. Thus, while C1 roots were the most affected in terms of vulnerability and lethal fire effect, the response of C2 roots to fire was a shift in production to where disturbance was of lower magnitude. Overall, only the C1 roots were affected by fire. Our findings agree with Hart et al.^58^, suggesting that different portions of plant organs might respond autonomously to changing local conditions. In particular, as in the case of drought or hypoxic conditions^59–61^, the interplay of fine root depth distribution, morphological traits, and branching orders may be a key functional response to stress determining plant survival and growth.

In our study, in the shallowest soil profile, the increase in length concurrent with a reduction in biomass might be a functional response to maximize soil exploitation (i.e., long root web through longitudinal growth) without costlier investments in large roots (i.e., low biomass). Moreover, our results concur with the general observations that fire reduced live fine-root dry weight of a pine, especially closer to the soil surface, 1 and 5 months after burning^13^.

At the broadest level (combining depths and root classes), fire significantly and more consistently increased dead root length and necromass compared to the control, but for live roots, which showed more nuanced and diverse results, these live root length and biomass remained unaffected. This trend was also reflected in the ratio of live-to-dead roots, where distinct fire-induced differences for C1 roots were apparent and increased with depth but not at the broadest level. Fire was more consistent with mortality and more consistent with root class, especially at the shallowest depth, resulting in short-term reductions in live root length and biomass. Even so, for the entire sampling time, fire was associated with increased fine-root productivity coupled with faster rate of turnover. The increase in fine root productivity following fire agrees with others in mixed boreal conifer‒broad-leafed forest stands^11,62^.

Morphological traits such as length, diameter, and biomass are important indicators of the foraging strategy, physiology, and lifespan of a root^63^. For example, under drier soil conditions, plants produce longer and finer roots^49,64^, which results in a relatively greater length per unit mass thereby leading to an increase in specific root length (SRL)^65^. Indeed, root length is assumed to be proportional to resource acquisition (benefit) and root mass to be proportional to construction and maintenance (cost)^66^. Thus, SRL is a good indicator of the benefit/cost analysis^49,66^. In our study, the effect of fire was significant for SRL only with C1 roots; burned trees had lower values. This significant effect transcended depth as well, suggesting that the finest roots of control trees were functioning more for resource acquisition than those of their fire-affected cohorts. On the one hand, it may be that C1 roots on fire-affected trees have greater access to fire-released nutrients and therefore have less need for morphology that supports foraging^11,62^. On the other hand, because the more ephemeral C1 roots are the thinnest and longest of the whole fine root population, they are more susceptible to heat-induced mortality; their mortality reduced the total fine root length at a higher magnitude than the biomass (i.e., more related to the thicker root fraction), thereby resulting in a general reduction of the SRL.

Root research is difficult, so seeing that the literature often presents fine root data at broader scales (i.e., < 2 mm) without segregation of live and dead roots, with few sampling times, and without soil depth differentiation (i.e., 30 cm depth) is unsurprising. Given our results, this lack of fine-resolution data may help explain contradictions in the literature, especially for biomass shown to either increase [tallgrass prairie;^67^], slightly decrease [tropical savanna;^68^], or remain constant [Brazilian savanna;^69^] after fire.

Changes in climate and resulting changes to disturbance regimes will test the resilience of current forests^70^. For trees, specific functional traits for adapting to climate change include those associated with rooting^71^, and our work shows that resilience may reside with the finest of the fine roots, which respond the most dynamically to disturbance. In support of the hypothesized fine-root spatial modulation, findings highlighted that beech trees in the southern slope of Italian Pre-Alps have an ability to change fine root growth distribution and architecture in response to wildfire occurrence, compensating for the damaged fine roots in the shallow soil layer by enhancing new high-order fine root growth at deeper soil depths where the magnitude of stress was lower. These traits unveil functional plasticity that may be a resilience mechanism to fire-derived stress^72^.

## Supplementary Information

Supplementary Information.

## Data Availability

The data presented in this study are available on request from the corresponding author.
