# Supplementary material for: Unusual late-fall wildfire in a pre-Alpine Fagus sylvatica forest reduced fine roots in the shallower soil layer and shifted very fine-root growth to deeper soil depth
Source: Sci Rep. 2023 Apr 19;13:6380. doi: 10.1038/s41598-023-33580-7 (PMC10115845; doi:10.1038/s41598-023-33580-7)
Supplement: Supplementary file 1 — Supplementary Information. [file 41598_2023_33580_MOESM1_ESM.pdf]

**Supplementary Table S1.** Hierarchical linear regression (HLS) model outputs for each fine root diameter class dependent variables (live root length, live root biomass, dead root length, necromass, and live specific root length). Sampled tree and soil core were used as grouping levels.

| | Root diameter (mm) | | |
|---|---|---|---|
| | Class 1 | Class 2 | Class 3 |
| Variables | 0–0.3 | 0.3–1.0 | 1.0–2.0 |
| **Live length** | | | |
| Cook's distance MAX | 0.044 | 0.048 | 0.056 |
| STD residual MIN | -3.38 | -3.53 | -3.59 |
| STD residual MAX | 2.44 | 2.24 | 1.46 |
| R squared | 0.151 | 0.128 | 0.137 |
| R squared change | 0.150 | 0.123 | 0.135 |
| % R squared variation | 0.66 | 3.91 | 1.46 |
| | | | |
| **Live biomass** | | | |
| Cook's distance MAX | 0.022 | 0.027 | 0.028 |
| STD residual MIN | -2.63 | -2.24 | -2.62 |
| STD residual MAX | 2.95 | 3.17 | 3.23 |
| R squared | 0.187 | 0.193 | 0.238 |
| R squared change | 0.184 | 0.189 | 0.236 |
| % R squared variation | 1.60 | 2.07 | 0.84 |
| | | | |
| **Dead length** | | | |
| Cook's distance MAX | 0.039 | 0.031 | 0.051 |
| STD residual MIN | -3.51 | -3.32 | -3.13 |
| STD residual MAX | 2.08 | 2.12 | 1.83 |
| R squared | 0.231 | 0.195 | 0.101 |
| R squared change | 0.227 | 0.191 | 0.097 |
| % R squared variation | 1.73 | 2.05 | 3.96 |
| | | | |
| **Necromass** | | | |
| Cook's distance MAX | 0.046 | 0.020 | 0.020 |
| STD residual MIN | -2.45 | -2.54 | -3.64 |
| STD residual MAX | 3.86 | 3.23 | 3.67 |
| R squared | 0.141 | 0.135 | 0.087 |

| | | | |
|---|---|---|---|
| R squared change | 0.139 | 0.133 | 0.08 |
| % R squared variation | 1.42 | 1.48 | 2.30 |
| | | | |
| **Specific root length** | | | |
| Cook's distance MAX | 0.039 | 0.038 | 0.038 |
| STD residual MIN | -3.74 | -2.97 | -3.89 |
| STD residual MAX | 3.73 | 3.28 | 2.74 |
| R squared | 0.090 | 0.064 | 0.035 |
| R squared change | 0.089 | 0.060 | 0.033 |
| % R squared variation | 1.11 | 6.25 | 5.71 |

**Supplementary Table S2.** Mean (± standard error) values for root traits by diameter class (C1: 0–0.3; C2: 0.3–1.0; C3: 1.0–2.0 mm; and all root classes combined: 0–2.0 mm) and soil depth. Mean values in **bold** within each root trait × soil depth combination represent significant differences (LSD tests) between control (C; n=40) and fire-affected (F; n=30) trees; *p* = *p* value.

| Diameter class | Soil depth (cm) | Standing biomass (g·m$^{-2}$) | | | Standing necromass (g·m$^{-2}$) | | | Live length (m·m$^{-2}$) | | | Dead length (m·m$^{-2}$) | | |
|---|---|---|---|---|---|---|---|---|---|---|---|---|---|
| | | C | F | *p* | C | F | *p* | C | F | *p* | C | F | *p* |
| 0–0.3 | 0–10 | **13.5** ±1.0 | **8.0** ±0.7 | **<0.001** | **0.23** ±0.05 | **0.58** ±0.12 | **<0.001** | **1267** ±110 | **706** ±65 | **<0.001** | **20.2** ±4.4 | **50.0** ±9.6 | **<0.001** |
| | 10–20 | **8.4** ±0.6 | **6.6** ±0.4 | **0.048** | **0.11** ±0.02 | **0.29** ±0.06 | **0.036** | **760** ±59 | **549** ±37 | **0.025** | **10.0** ±1.7 | **25.8** ±5.2 | **0.032** |
| | 20–30 | 6.3 ±0.5 | 7.6 ±0.6 | 0.208 | **0.07** ±0.02 | **0.27** ±0.07 | **0.042** | 565 ±44 | 654 ±58 | 0.862 | **6.7** ±1.8 | **23.0** ±5.9 | **0.035** |
| 0.3–1.0 | 0–10 | **74.4** ±5.1 | **58.6** ±5.4 | **0.011** | **5.5** ±0.7 | **13.2** ±1.8 | **<0.001** | **805** ±60 | **606** ±55 | **0.003** | **39.0** ±5.3 | **100.1** ±15.6 | **<0.001** |
| | 10–20 | 55.2 ±3.3 | 66.9 ±5.2 | 0.061 | **5.6** ±0.8 | **11.2** ±1.5 | **0.001** | 553 ±34 | 657 ±53 | 0.115 | **35.0** ±4.9 | **77.5** ±12.0 | **0.001** |
| | 20–30 | **35.4** ±2.6 | **58.5** ±4.0 | **<0.001** | **2.8** ±0.6 | **7.6** ±1.2 | **0.005** | **363** ±26 | **587** ±41 | **0.001** | **18.3** ±3.3 | **57.7** ±11.0 | **0.005** |
| 1.0–2.0 | 0–10 | 33.9 ±3.5 | 38.4 ±4.4 | 0.439 | **7.0** ±1.3 | **13.4** ±2.3 | **0.002** | 41.4 ±4.3 | 43.5 ±4.8 | 0.746 | **8.8** ±1.6 | **15.0** ±2.4 | **0.007** |
| | 10–20 | 47.0 ±4.1 | 42.1 ±4.3 | 0.394 | 8.1 ±1.3 | 10.3 ±1.4 | 0.267 | 49.9 ±4.0 | 48.4 ±4.7 | 0.815 | 9.6 ±1.4 | 12.2 ±1.5 | 0.239 |
| | 20–30 | 34.2 ±3.9 | 36.1 ±4.5 | 0.750 | 5.1 ±0.9 | 6.6 ±1.1 | 0.455 | 37.3 ±4.3 | 42.0 ±5.2 | 0.462 | 6.1 ±1.2 | 8.4 ±1.3 | 0.334 |
| 0–2.0 | 0–30 | 317 ±21 | 341 ±39 | 0.785 | **32.8** ±6.0 | **55.9** ±12.6 | **0.002** | 4337 ±210 | 3915 ±216 | 0.165 | **138** ±15 | **288** ±40 | **<0.001** |

**Supplementary Table S3.** Mean (± standard error) values for root traits by diameter class (C1: 0–0.3; C2: 0.3–1.0; C3: 1.0–2.0 mm; and all root classes combined: 0–2.0 mm) and soil depth. Mean values in **bold** within each root trait × soil depth combination represent significant differences (LSD tests) between control (C; n=40) and fire-affected (F; n=30) trees; $p$ = $p$ value. Production values determined with decision matrix method. Turnover rate is the quotient of annual production and maximum standing biomass.

| Diameter class | Soil depth (cm) | Specific root length ($m \cdot g^{-1}$) | | | Live/dead mass | | | Production ($g \cdot m^{-2}$) | | | Turnover rate | | |
|---|---|---|---|---|---|---|---|---|---|---|---|---|---|
| | | C | F | $p$ | C | F | $p$ | C | F | $p$ | C | F | $p$ |
| 0–0.3 | 0–10 | **92.5 ±1.5** | **87.7 ±1.3** | **0.003** | **81.1 ±17.7** | **15.8 ±4.2** | **0.003** | 9.6 ±2.1 | 9.0 ±0.6 | 0.788 | **0.51 ±0.07** | **0.74 ±0.04** | **0.015** |
| | 10–20 | **89.9 ±1.1** | **83.9 ±0.9** | **<0.001** | **83.4 ±13.3** | **35.1 ±15.3** | **0.032** | 7.5 ±1.9 | 7.2 ±2.1 | 0.913 | 0.58 ±0.11 | 0.66 ±0.11 | 0.609 |
| | 20–30 | **89.1 ±0.9** | **85.9 ±1.1** | **0.039** | **101.6 ±19.1** | **45.7 ±15.9** | **0.044** | 9.4 ±2.2 | 12.0 ±1.8 | 0.381 | 0.85 ±0.12 | 0.90 ±0.13 | 0.781 |
| 0.3–1.0 | 0–10 | 10.9 ±0.2 | 10.6 ±0.2 | 0.245 | **16.0 ±3.7** | **5.2 ±1.5** | **0.016** | 95.5 ±13.3 | 79.9 ±7.2 | 0.328 | 0.85 ±0.08 | 0.93 ±0.10 | 0.558 |
| | 10–20 | 10.2 ±0.2 | 9.8 ±0.2 | 0.244 | 10.5 ±2.0 | 8.2 ±2.8 | 0.533 | 56.0 ±8.5 | 97.6 ±28.4 | 0.191 | 0.77 ±0.13 | 0.80 ±0.14 | 0.891 |
| | 20–30 | 10.5 ±0.2 | 10.2 ±0.2 | 0.346 | 14.5 ±2.8 | 9.4 ±1.2 | 0.103 | 59.3 ±10.0 | 75.0 ±16.3 | 0.433 | 1.04 ±0.14 | 0.79 ±0.11 | 0.186 |
| 1.0–2.0 | 0–10 | 1.29 ±0.04 | 1.21 ±0.04 | 0.090 | 4.7 ±1.4 | 4.4 ±1.6 | 0.882 | 76.3 ±12.6 | 72.4 ±9.0 | 0.806 | 1.35 ±0.10 | 1.26 ±0.21 | 0.777 |
| | 10–20 | 1.15 ±0.03 | 1.24 ±0.03 | 0.101 | 9.4 ±2.8 | 5.6 ±1.8 | 0.269 | 64.9 ±11.7 | 100.6 ±25.4 | 0.221 | 0.81 ±0.10 | 1.40 ±0.31 | 0.095 |
| | 20–30 | 1.21 ±0.04 | 1.24 ±0.03 | 0.331 | 11.2 ±3.7 | 6.7 ±1.1 | 0.265 | 66.8 ±9.8 | 89.1 ±15.2 | 0.244 | 1.14 ±0.08 | 1.33 ±0.09 | 0.145 |
| 0–2.0 | 0–30 | 14.3 ±0.6 | 12.7 ±0.6 | 0.203 | 5.3 ±1.4 | 2.9 ±1.0 | 0.428 | 444.4 ±36.5 | 542.8 ±67.7 | 0.305 | 1.07 ±0.07 | 1.29 ±0.11 | 0.106 |
